# Effects of soil water on fungal community composition along elevational gradients on the northern slope of the Central Kunlun Mountains

**DOI:** 10.3389/fmicb.2024.1494070

**Published:** 2025-01-08

**Authors:** Yongguang Zhang, Chaonan Li, Zhihao Zhang, Chenhong Li, Bo Zhang, Hongchen Jiang, Waqar Islam, Xiangzhen Li, Fanjiang Zeng

**Affiliations:** ^1^Faculty of Agriculture, Forestry and Food Engineering, Yibin University, Yibin, China; ^2^Xinjiang Key Laboratory of Desert Plant Roots Ecology and Vegetation Restoration, Xinjiang Institute of Ecology and Geography, Chinese Academy of Sciences, Urumqi, China; ^3^Ecological Security and Protection Key Laboratory of Sichuan Province, Mianyang Normal University, Mianyang, China; ^4^State Key Laboratory of Biogeology and Environmental Geology, China University of Geosciences, Wuhan, China; ^5^Engineering Research Center of Soil Remediation of Fujian Province University, College of Resources and Environment, Fujian Agriculture and Forestry University, Fuzhou, China

**Keywords:** soil ecosystem, elevational gradients, Central Kunlun Mountains, driving factors, ecological processes

## Abstract

Soil fungi are essential to ecosystem processes, yet their elevational distribution patterns and the ecological mechanisms shaping their communities remain poorly understood and actively debated, particularly in arid regions. Here, we investigated the diversity patterns and underlying mechanisms shaping soil fungal communities along an elevational gradient (1,707–3,548 m) on the northern slope of the Central Kunlun Mountains in northwest China. Results indicated that the dominant phyla identified across the seven elevational gradients were *Basidiomycota* and *Ascomycota*, displaying a unimodal pattern and a U-shaped pattern in relative abundance, respectively. Soil saprotroph and nectar/tap saprotroph were the dominant functional groups (>1.0%). Along the elevational gradients, soil fungal α-diversity demonstrated a generally decreasing trend, whereas β-diversity showed a contrasting increasing trend. Among the environmental variables, altitude and climate (mean annual precipitation, MAP; mean annual temperature, MAT) were the strongest predictors for α-diversity. Partial least squares path modeling (PLSPM) analysis revealed that soil water content (Wat) was the most influential factor driving fungal α-diversity, while vegetation coverage (Veg) emerged as the primary determinant of soil fungal community composition. The influence of Wat on fungal α-diversity shifted from indirect to direct as elevation increased, transitioning from lower elevations (≤2,448 m) to higher elevations (≥2,746 m). Similarly, the impact of Veg on soil fungal community composition exhibited a comparable pattern. The null model analysis revealed that homogeneous selection and dispersal limitation dominated the soil fungal community assembly at elevations lower than 2,448 m and higher than 2,746 m, respectively. Variations in ecological processes may be linked to changes in key environmental factors that influence soil fungal communities in an elevation-dependent manner. These findings can enhance our ability to predict soil fungal diversity patterns and their responses to climate change in the ecosystems of the northern slope of the Central Kunlun Mountain.

## 1 Introduction

Soil fungi play vital ecological roles in terrestrial ecosystems (Frac et al., 2018). For example, a significant group of soil mycorrhizal fungi forms symbiotic relationships with plant roots, assisting their hosts in acquiring nutrients in exchange for photosynthetically derived carbon (Shi et al., 2023; van der Heijden et al., 1998). Some soil fungi play a crucial role as primary agents in the decomposition of plant litter (Liers et al., 2011). In the last decade, internal transcribed spacer (ITS) regions have been used as biomarkers to study the fungal diversity and biogeographic distribution. Based on correlations between fungal taxa and their potential ecological functions inferred from genomic analyses and laboratory studies, soil fungi are categorized into distinct functional guilds (Nguyen et al., 2016; Treseder and Lennon, 2015; Põlme et al., 2020). Climate changes greatly affect the richness, composition, and ecological processes of soil fungi (Jansson and Hofmockel, 2019; Peay et al., 2016; Mikryukov et al., 2023). Understanding the changes in soil fungal community diversity and the underlying mechanisms in response to global climate change is crucial. This knowledge will enhance our comprehension of how climate change affects soil biota and the ecosystem services they provide (Jansson and Hofmockel, 2019; Graham et al., 2016).

Several abiotic and biotic factors influence the diversity and distribution patterns of soil fungal communities (Tedersoo et al., 2014; Mikryukov et al., 2023). Water availability plays a critical role in regulating soil biodiversity and function (Zhang et al., 2023; Schimel, 2018). Despite its multifaceted influence, contrasting perspectives exist regarding the role of water in shaping the diversity and composition of soil fungal communities. On one hand, water is considered the most influential factor. Studies have shown that increasing aridity significantly reduces soil fungal diversity and abundance in global drylands (Maestre et al., 2015). Similarly, research in arid regions of northwest China identified aridity as the primary environmental factor affecting fungal richness, β-diversity, and species co-occurrence across various ecosystems, including agricultural fields, forests, wetlands, grasslands, and deserts (Jiao et al., 2021). On the other hand, some studies suggest that climate, rather than water availability, is the dominant factor shaping soil fungal composition (Mikryukov et al., 2023; Tedersoo et al., 2014). To resolve these contrasting views, further studies are necessary to provide deeper insights into the relative roles of water and climate in structuring soil fungal communities. Altitude is one of the most important gradients with many concentrated environmental variables within a short distance (Körner, 2007). Climate zones often shift with increasing altitude over shorter distances compared to horizontal spatial gradients. Similar patterns are observed for edaphic properties and vegetation, making mountain systems excellent natural laboratories for studying biodiversity responses and ecosystem processes under global climate change (Sundqvist et al., 2013). Species diversity often exhibits regular changes along elevational gradients (Wang et al., 2022; McCain and Grytnes, 2010). For soil fungi, several diversity patterns have been reported, including increasing trends (Ogwu et al., 2019), decreasing patterns (Shen et al., 2020), hump-shaped patterns (Huang et al., 2023), U-shaped patterns (Li et al., 2022), and no significant trend (Liu et al., 2018). Both abiotic factors (Tian et al., 2017; Shen et al., 2014; Liu et al., 2018; Shen et al., 2020) and biotic interactions (Yang et al., 2017) have been identified as major drivers shaping soil fungal community structure along these gradients.

Understanding the ecological processes that shape microbial diversity patterns is a fundamental topic in ecology (Hanson et al., 2012). Microbial community assembly is primarily influenced by deterministic and stochastic processes (Stegen et al., 2013, 2012). Deterministic processes include factors such as variable selection and homogeneous selection, whereas stochastic processes involve dispersal limitation and homogenizing dispersal (Stegen et al., 2015). Along elevational gradients, deterministic processes often dominate as evidenced by stronger phylogenetic clustering in microbial communities (Wang et al., 2022, 2012). However, contrasting views exist regarding soil fungi, where stochastic processes are suggested to play a larger role in community assembly. For instance, Hussain et al. (2021) reported that stochastic processes dominated soil fungal community assemblages across elevations (889–3,837 m). Similarly, Wang et al. (2023) found that stochastic processes primarily shaped fungal communities in forest and grassland soils. Despite these findings, few studies have focused specifically on the ecological processes driving soil fungal communities (Wang et al., 2023; Mikryukov et al., 2023; Hussain et al., 2021; Jiao et al., 2021). The contrasting perspectives highlight the need for more evidence to clarify the ecological mechanisms governing soil fungal community assembly. Arid lands host a significant proportion of global soil microbes, with unique adaptation mechanisms that enable survival in harsh environments (Pointing and Belnap, 2012). Compared to other regions, arid lands are more vulnerable to climate change, particularly the intensification of drought (Maestre et al., 2015, 2016). The Kunlun Mountains have become a focal point for studying species richness and biogeographical distribution patterns of biodiversity (Du et al., 2022). On the northern slope of the Central Kunlun Mountains, a diverse array of plants thrives, despite the challenging climate at the intersection of the temperate continental desert and the extremely cold-arid climate of the Qinghai–Tibet Plateau. Plant species diversity in this area shows a monotonically increasing trend with altitude, ranging from 1,960 to 4,100 m (Gui et al., 2010). Plants influence soil biota primarily through root exudation and litter deposition (Vives-Peris et al., 2020; Waldrop et al., 2006), which serve as vital resources for soil systems.

Based on the above observations, we hypothesized that: (1) soil water availability would be the primary factor shaping the richness and composition of soil fungal communities in mountain systems; (2) above-ground vegetation would significantly affect soil fungal diversity; (3) stochastic processes would dominate the assembly of soil fungal communities on the northern slope of the Central Kunlun Mountains. This study aimed to address three key questions: (1) How do the diversity patterns of soil fungal communities change along elevational gradients? (2) Which factors shape these diversity patterns along the gradients? (3) What ecological processes primarily structure the soil fungal communities? Our findings will provide new insights into the elevational diversity patterns of soil fungi and their responses to climate change, contributing to the design of biodiversity conservation strategies for ecologically fragile regions globally.

## 2 Material and methods

### 2.1 Soil sampling

The study area was located on the northern slope of the Central Kunlun Mountains. Seven altitudes (1,707, 1,960, 2,448, 2,746, 2,905, 3,248, and 3,548 m) along the Cele Valley were selected as the sampling sites. The mountain–oasis–desert observation transects were established by the Xinjiang Institute of Ecology and Geography, Chinese Academy of Sciences with the aim to study the biodiversity patterns and their responses to global climate changes. The slopes of the mountain range exhibit significant variations in climate, causing pronounced shifts in vegetation cover due to the steep climate gradient (Du et al., 2022). With the increase in elevational gradients, vegetation coverage (Veg) gradually increased from 0.5 to 85.0%, mean annual precipitation (MAP) increased from 62.7 mm at 1,707 m to 362.1 mm at 3,548 m, whereas mean annual temperature (MAT) decreased from 11.2 to 1.2°C. All climate data were collected from climate stations (HOBO U30, ONSET, USA) at each elevation site during the period from 2019 to 2020.

Soil samples were collected in September 2018 at the end of summer. At each site, five independent quadrats (2 m × 2 m) were established at 15-m horizontal intervals. From each quadrat, five topsoil cores (0–10 cm) were collected from four corners and the center after removing the litter. These cores were then mixed evenly to form a single composite sample. In total, 35 soil samples were collected from quadrats at seven different elevational gradients. During sampling, the soil drill was cleaned to remove any residual soil and disinfected with 75% alcohol before collecting each sample. After returning to the laboratory, soil samples were passed through a sterilized 2-mm sieve and dried. Each sample was then split into two portions: one was frozen at −80°C for environmental DNA extraction, and the other was dried for physical and chemical parameter analysis.

### 2.2. Determination of soil physical and chemical parameters

Soil water content (Wat) was assessed by oven drying to constant weight at 105°C. Soil pH and electrical conductivity (EC) were determined using pH and conductivity meters after a soil: purified water (1:5) suspension. Total water-soluble salt in soil (Sal) was determined by the residue drying-quality method. Total nitrogen (TN) was measured using the Kjeldahl method. Total organic carbon (TOC) was measured with the K_2_Cr_2_O_7_ oxidation method. ${\text{NH}}_{4}^{+}$-N and ${\text{NO}}_{3}^{-}$-N contents were extracted with 1M KCl and then determined with the ultraviolet spectroscopy method. Available phosphorus (AP) was assessed with the molybdate/ascorbic acid method.

### 2.3. Soil DNA extraction, fungal ITS amplification, and sequencing

Genomic DNAs were extracted from 0.25 g fresh soil using MO BIO powerful soil DNA extraction kit (MO BIO Laboratories, Carlsbad, CA, USA). The quality and concentration of the DNA extracted were evaluated using a NanoDrop ND-1000 spectrophotometer (Thermo Fisher Scientific, Waltham, MA, USA). High-quality DNA was used for the polymerase chain reaction (PCR) amplification. The primers ITS4R (5′-TCCTCCGCTTATTGATATGC-3′) and gITS7F (5′-GTGARTCATCGARTCT TTG-3′) were used to amplify the gene fragments of 5.8S RNA and large subunit RNA. The amplification conditions were 94°C 3 min, 94°C 40 s, 56°C 60 s, and 72°C 60 s, with a total of 30 cycles and the last 72°C 10 min. All PCR products were checked by using a NanoDrop ND-1000 Spectrophotometer, pooled at equal molar amounts for each soil sample. Then, the qualified PCR amplification products were sequenced by using the Illumina NovaSeq 6000 sequencing platform (Biobit Biotech Inc., Chengdu, China). Raw ITS sequences were deposited into NCBI with an accession number of PRJNA1158445.

### 2.4. Bioinformatics analysis

Paired-end sequences were spliced with FLASH (V 1.2.7 http://ccb.jhu.edu/software/FLASH/) (Magoč and Salzberg, 2011). The quality of the obtained Raw Tags was strictly filtered by QIIME 1.9.1 (http://qiime.org/scripts/split_libraries_fastq.html) (Caporaso et al., 2010). Briefly, the Raw Tags were truncated from the first low-quality base site with a continuous low-quality value (≤3) to the default length of the base number (3). The Tags data set obtained was further filtered by removing the continuous high-quality base length <75% of the Tags length. Then, the chimeric sequences were detected with Usearch software (v8.0, http://drive5.com/uparse/) (Edgar, 2013), and the chimeras were removed. The obtained effective Tags were clustered into operational taxonomic units (OTU) with 97.0% similarity by default with QIIME (Caporaso et al., 2010), and the detected Singleton was removed. The representative OTUs were taxonomically annotated using a classify-sklearn algorithm with UNITES version 10.0 as the reference database (Abarenkov et al., 2024). We rarefied the sequence number to 2,948 for each sample to eliminate the difference in sequencing depth among samples (Supplementary Figure S1). Then, a phylogenetic tree was constructed using the QIIME 1.9.1 built-in tools. Fungal functional guilds were inferred using the microeco R package v0.1.2 (Liu et al., 2021) by comparing taxonomic data with the FungalTraits database (v1.2) (Põlme et al., 2020).

### 2.5 Statistical analysis

The observed OTUs (α-diversity) and Bray–Curtis distances (β-diversity) were calculated using the built-in functions of the microeco package (Liu et al., 2021). Soil characteristics, climate data, vegetation coverage, and observed OTUs were compared across altitudes using the Wilcoxon rank-sum test with a false discovery rate (FDR) threshold of <0.005. Significant taxa (biomarkers) across elevational gradients were identified using a non-parametric test combined with linear discriminant analysis (LDA) (Liu et al., 2021). Analysis of similarity (ANOSIM) was employed to assess differences in environmental variables (Euclidean distance) and soil fungal communities (Bray–Curtis distance), while principal coordinate analysis (PCoA) was used for visualization. Distance-based redundancy analysis (dbRDA) was performed to quantify the explanatory values for fungal community composition and visualized using the ggplot2 package. The significance of dbRDA results was tested with the anova.cca function in the vegan R package. Indices such as the standardized effect size measure of the mean nearest taxon distance (ses.MNTD) and the β-nearest taxon index (βNTI) were used to evaluate phylogenetic community assembly (Stegen et al., 2012). The ses.MNTD quantifies deviations from the mean nearest taxon distance in null distributions, with values <-2 indicating phylogenetic over-dispersion and >+2 suggesting phylogenetic clustering (Webb et al., 2002). Turnover in phylogenetic composition was quantified using βNTI, where values >+2 or <-2 indicate assembly processes significantly differing from null expectations (Stegen et al., 2012). To explore the influence of ecological processes on soil fungal communities across elevations, ses.MNTD, βNTI, and community assembly processes were analyzed with the iCAMP R package (Ning et al., 2020). Differences in α-diversity, β-diversity, ses.MNTD, and βNTI across altitudes were assessed using Wilcoxon rank-sum tests (FDR <0.05). Spearman correlations, adjusted with the Holm method (psych R package), were used to link the Shannon–Weaver index, Bray–Curtis distances, ses.MNTD, and βNTI with key environmental factors. The relative importance of geographic and environmental factors in shaping fungal communities was evaluated using multiple regression on matrices (MRM) due to its robustness (Lichstein, 2007). Partial least squares path modeling (PLSPM; plspm R package) was applied to illustrate the hierarchical effects of environmental changes on the fungal Shannon–Weaver index and community composition (PCoA axes). High collinearity among environmental variables was addressed using the varclus function in the Hmisc package, with a Spearman's ρ^2^ threshold of >0.8. All statistical analyses and visualizations were performed using R v4.3.2.

## 3 Results

### 3.1 Changes of environmental variables along the elevational gradients

Across the whole transect, environmental variables changed regularly (Supplementary Figure S2). MAP and Wat showed increased trends with the highest values at 3,548 m. Instead, MAT decreased with the increase in the elevational gradients. Soil pH values exhibited a unimodal trend with the highest value at 2,905 m. As for soil nutrients, TOC and TN increased across the whole altitude range except that the contents of the two variables got the lowest values at 2,746 m. However, the highest ratio of TOC to TN (C_N) was observed at 1,960 m. Available nitrogen including ${\text{NO}}_{3}^{-}$-N and ${\text{NH}}_{4}^{+}$-N gave U-shaped curves with the lowest contents at 2,746 m. AP changed complexly with a hump (peak at 2,448 m), then an increasing trend from 2,746 to 3,548 m. EC and Sal exhibited almost similar trends. Veg showed an almost increased trend with the highest value at 3,248 m. The anosim test analysis indicated that the comprehensive environmental variables significantly differed between the different sampling sites (*P* < 0.05) (Supplementary Table S1). Moreover, the result was also confirmed by PCoA based on Euclidean distance (Supplementary Figure S3).

### 3.2 Differences of the soil fungal composition along the elevational gradients

**Figure 1:**
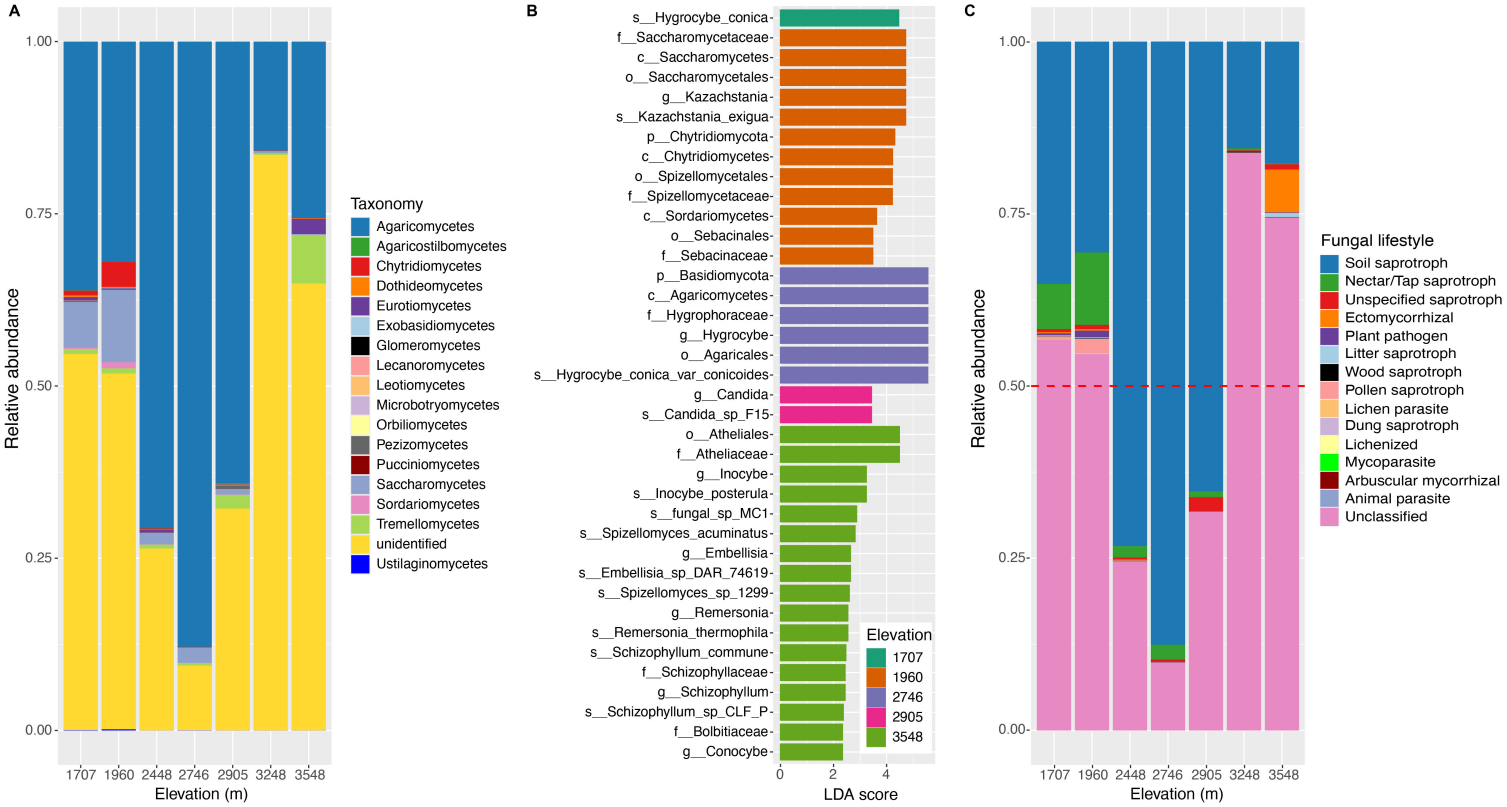
Composition of soil fungal community at the class level and fungal functional guilds along the elevational gradients on the northern slope of the Central Kunlun Mountains. **(A)** Relative abundances of the dominant fungal classes in the soil fungi community; **(B)** different biomarkers among the different altitudes based on linear discriminant analysis (LDA); **(C)** the relative abundances of the fungal functional guilds in the soil samples collected from the different elevations.

A total of 1,978,086 validated high-quality sequences were obtained from the elevational soil samples, of which 2,948 OTUs were identified. Based on the UNITE 10.0 database, ~67% of sequences were classified into five phyla across all study sites, among which *Basidiomycota* and *Ascomycota* were the predominant phyla with relative abundances of 50 and 14.3%, respectively (Supplementary Figure S4). The other three phyla including *Chytridiomycota, Glomeromycota*, and *Zygomycota* only accounted for ~2.7% of total identified sequences. The relative abundance of *Basidiomycota* exhibited a hump-shaped pattern along the elevational gradients, while that of *Ascomycota* was like a U-shaped pattern (Supplementary Figure S4). At the class level, ~53.9% of the total sequences were classified. The relative abundance of the classes classified showed a complex trend (Figure 1A). Along the elevational gradients of the Central Kunlun Mountain, the prevalence of the first class *Agaricomycetes* showed a hump-shaped pattern with the peak at 2,746 m and an increase at 3,548 m. The prevalence of *Saccharomycetes* was observed across the elevational gradients, and its relative abundance exhibited a hump-shaped pattern with a turning point at 1,960 m. As for *Tremellomycetes*, its relative abundance showed an almost increased pattern. The prevalences of *Chytridiomycetes* and *Sordariomycetes* also exhibited a hump-shaped pattern with a peak at 1,960 m. A similar pattern occurred for that of *Pezizomycetes* with its peak at 2,905 m. However, the prevalence of *Eurotiomycetes* showed a complex pattern. As described, the relative abundance of different classes showed different patterns and were taxon-dependent.

LDA analysis identified distinct ecological biomarkers (LDA >4, *P* < 0.05) in the soil fungal communities at 1,707, 1,960, 2,746, 2,905, and 3,548 m (Figure 1B). The site at 3,548 m showed the highest enrichment with 17 biomarkers, followed by 1,960 m with 12 biomarkers. In contrast, 1,707, 2,746, and 2,905 m had 1, 6, and 2 biomarkers, respectively, whereas no biomarkers were detected at 2,448 and 3,248. These findings reveal clear elevational patterns in the composition and relative abundance of soil fungal communities along the northern slope of the Central Kunlun Mountains.

### 3.3 Differences in the composition of fungal functional guilds along the elevational gradients

Partitioning soil fungal communities by lifestyle revealed 2,948 OTUs classified into 14 functional guilds, with 1,454 OTUs unassigned to any known guild. Soil saprotrophs dominated, representing 88.4% of all OTUs, peaking at 2,746 m and following a unimodal pattern along the elevation gradient (Figure 1C). Nectar/tap saprotrophs and pollen saprotrophs also showed unimodal patterns, peaking at 1,960 m. Conversely, unspecified saprotrophs were most prevalent at 2,905 m, and ectomycorrhizal fungi peaked at 3,548 m, exhibiting a U-shaped pattern (Figure 1C). Other guilds, including plant pathogens, litter saprotrophs, and mycoparasites, constituted minor portions of the soil fungal communities across all elevations (Figure 1C).

### 3.4. Fungal diversity changes along the elevational gradients

**Figure 2:**
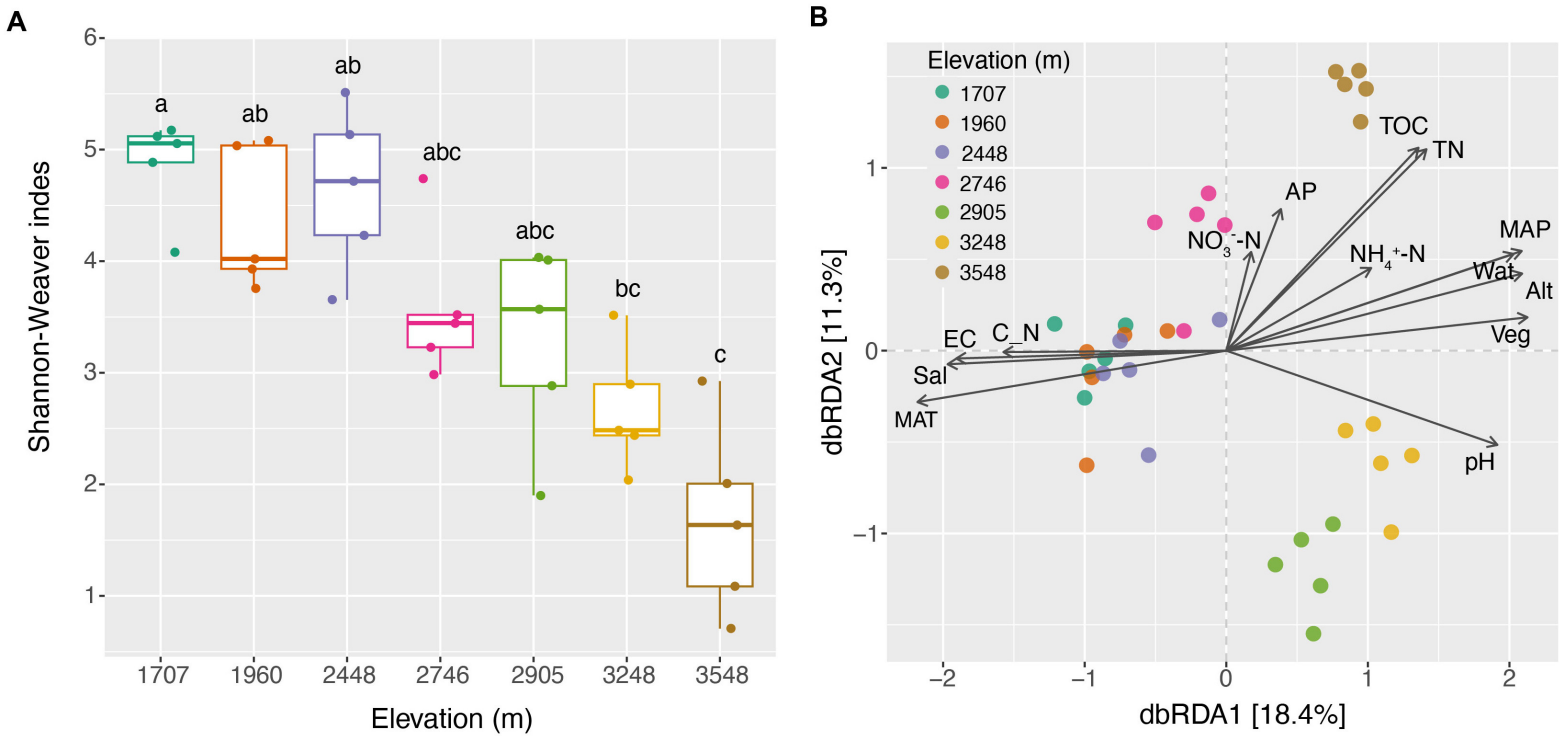
Diversity changes of fungal community along the elevational gradients and their relationships with the environmental factors. **(A)** Differences of Shannon–Weaver indexes; **(B)** effects of the environmental factors on the composition of soil fungal community based on dbRDA analysis. Alt, altitude; AP, available phosphorus; C_N, the ratio of total organic carbon to total nitrogen; EC, electrical conductivity; MAP, mean annual precipitation; MAT, mean annual temperature; Sal, total water-soluble salt; TN, total nitrogen; TOC, total organic carbon; Veg, vegetation coverage; Wat, water content.

Kruskal–Wallis–Dunn's test indicated that the fungal Shannon–Weaver Index exhibited no consistent changes along the elevational gradients, but did show a significant decrease with the increase of elevations (Figure 2A). PCoA showed that the compositions of soil fungal community structure were elevation-dependent. Fungal communities of the elevations 2,905, 3,248, and 3,548 m were distributed in separate regions, whereas those of the other four altitudes were gathered in one bigger region. ANOSIM analysis showed that fungal diversity based on Bray–Curtis distance exhibited a significant increase (*P* < 0.05) trend between the elevations of 1,707 and 2,746, 1,707 and 3,548, and 1,960 and 3,548 m. Totally, fungal diversity based on Bray–Curtis distance exhibited an almost increased trend with the increase of elevational gradients (Supplementary Table S2).

### 3.5 Effects of environmental variables on diversity and structure of soil fungal community

**Figure 3:**
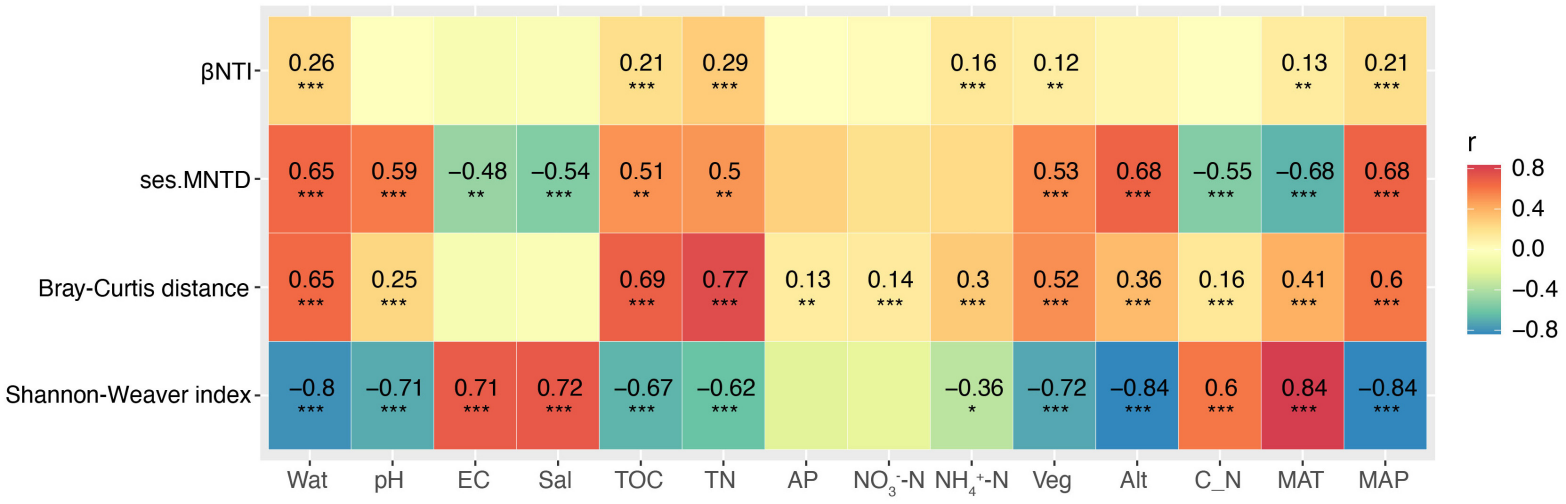
Spearman's correlation analysis of the relatedness between the environmental variables and the diversity, and phylogenetic diversity of soil fungal community. Alt, altitude; AP, available phosphorus; C_N, the ratio of total organic carbon to total nitrogen; EC, electrical conductivity; MAP, mean annual precipitation; MAT, mean annual temperature; Sal, total water-soluble salt; TN, total nitrogen; TOC, total organic carbon; Veg, vegetation coverage; Wat, water content. Significance level, *P < 0.05, **P < 0.01, ***P < 0.001, ns, not significant.

With the increase in elevational gradients, significant changes were observed in some environmental factors between the sites studied (Supplementary Figures S2, S3, Supplementary Table S1). The dbRDA analysis revealed the existence of a close correlation between the soil fungal community structure and the environmental factors (Figure 2B). Further permutation test indicated that Wat, pH, and Veg significantly correlated with the composition of soil fungal community (explained 52.4% of the total variance, *P* = 0.001; Supplementary Table S3). The effects of environmental variables on soil fungal communities changed with the increase of the elevational gradients (Supplementary Table S3). Spearman's correlation analysis indicated that Alt, MAP, and MAT significantly showed the highest correlated values with soil fungal α-diversity (Shannon–Weaver index), followed by some soil parameters and Veg (Figure 3). However, soil properties, followed by Veg, climate, and altitude significantly correlated with soil fungal β-diversity (Bray–Curtis distance; Figure 3).

**Figure 4:**
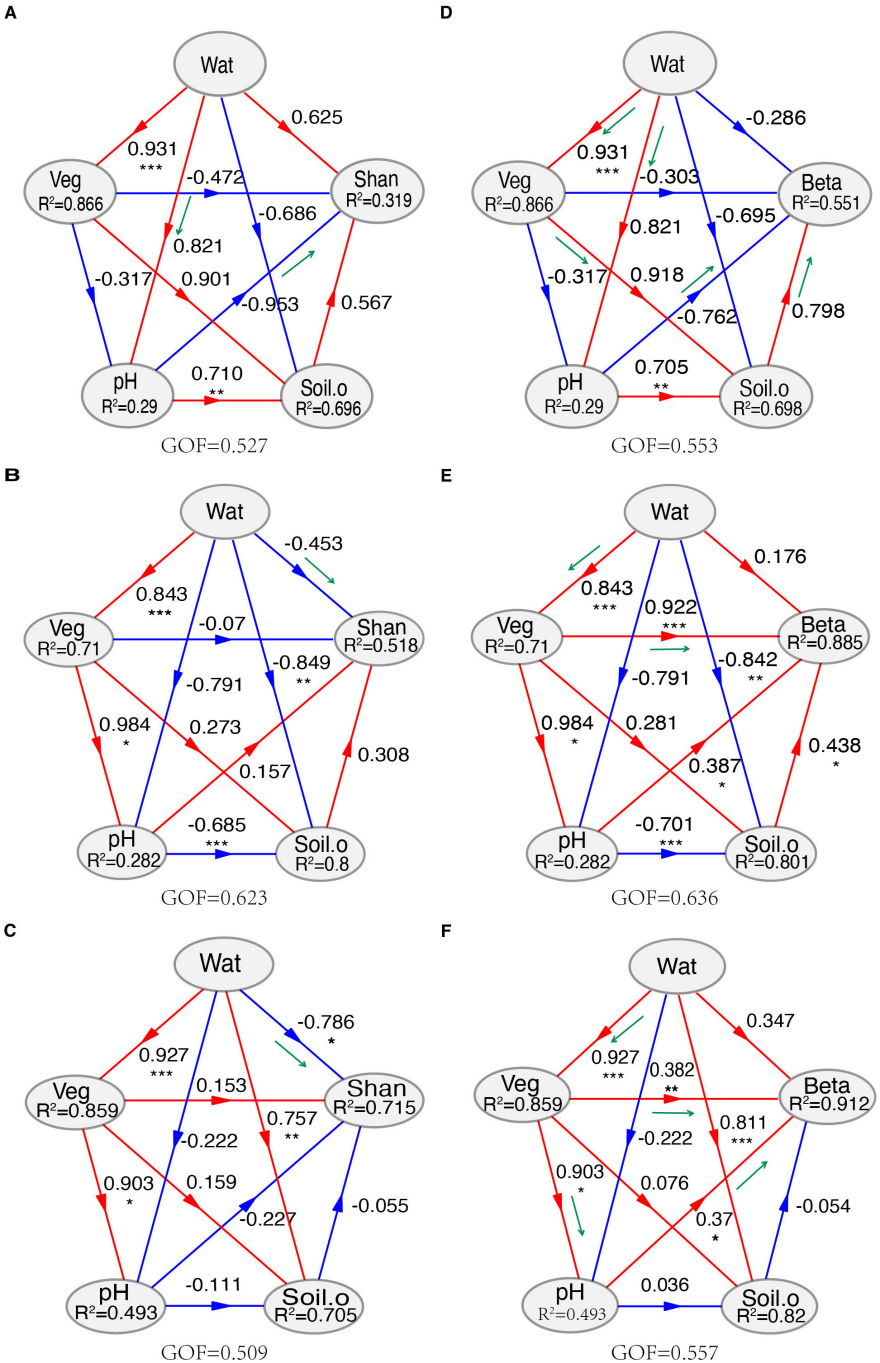
Partial least squares path models (PLSPMs) for the Shannon–Weaver index **(A–C)** and community composition **(D–F)**. **(A, D)** Lower elevations (1,707, 1,960, and 2,448 m); **(B, E)** higher elevations (2,746, 2,905, 3,248, and 3,548 m); **(C, F)**, the whole transect. The models were constructed at the conditions of the removal of some variables, which showed higher collinearities than 0.8 (Spearman's ρ^2^ > 0.8) calculated with the function varclus in the Hmisc package. The variables “Soil.o” include vary on the lower, higher, or whole transect. For the whole transect, “Soil.o” includes total nitrogen, TN; NH4^+^-N, ammonium nitrogen; NO3^−^-N, nitrate nitrogen; Sal, total water-soluble salt; AP, available phosphorus; Veg, vegetation coverage; Wat, water content. Shan, Shannon–Weaver index; Beta, the first two axes of PCoA. GOF: goodness of fit. Loading values of environmental for the modules in partial least squares path modeling (PLSPMs) are available in Supplementary Table S5. Significance level, *P < 0.05, **P < 0.01, ***P < 0.001, ns, not significant.

MRM analysis indicated that TN was the strongest factor correlated with the composition of soil fungal community, followed by TOC, Wat, and other factors. Furthermore, the effects of environmental factors and geographic distance on the composition of soil fungal communities differed greatly at the lower and the higher elevational gradients (Supplementary Table S4). PLSPMs revealed that Wat mainly shaped the species diversity (Shannon–Weaver index) of the soil fungal community, while Veg was the most important factor, followed by pH, in structuring the fungal community (Figure 4, Supplementary Table S5). With increasing elevation, the influence of Wat on the fungal Shannon–Weaver index shifted from an indirect to a direct effect, corresponding to the transition from lower elevations (≤2,448 m) to higher elevations (≥2,746 m). The effect of Veg on the composition of soil fungal community showed a similar way (Figure 4, Supplementary Table S5).

### 3.6. Ecological processes underlying soil fungal community along elevational gradients

**Figure 5:**
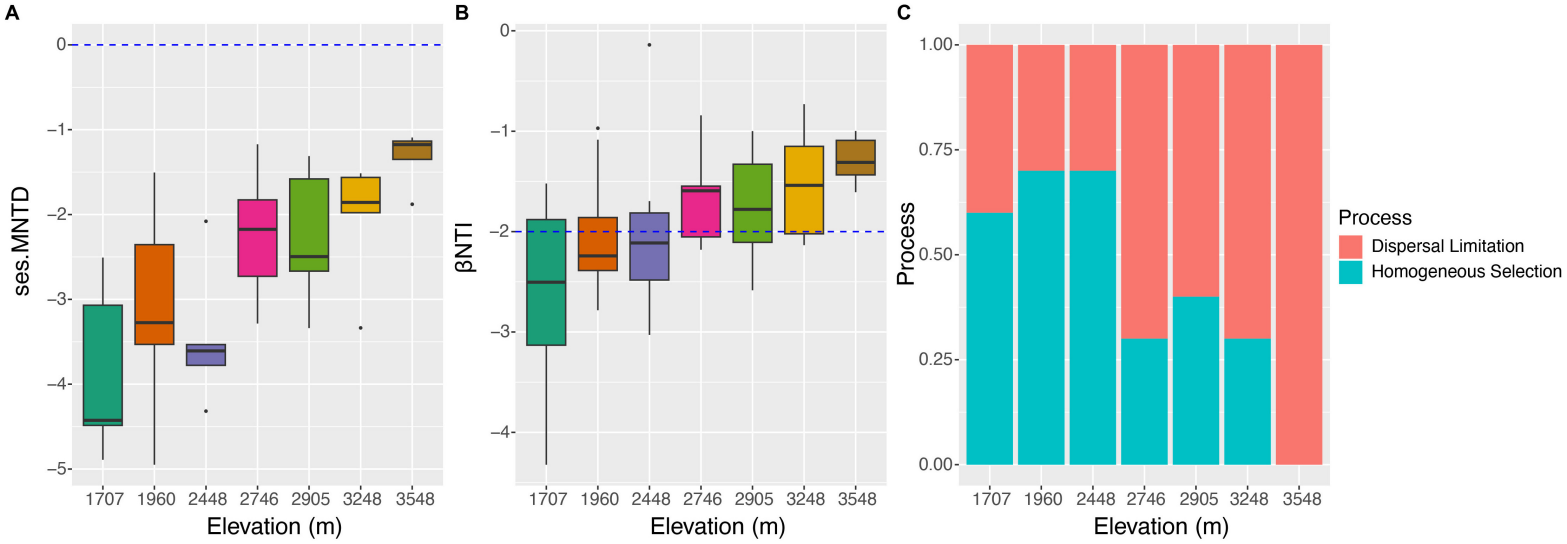
Profiles of phylogenetic diversities and the ecological processes of soil fungal community along the elevational gradients. **(A)** Differences of the values of ses.MNTD; **(B)** difference of the values of βNTI; **(C)** effects of ecological processes on the soil fungal community construction.

Phylogenetic null model analysis revealed that the median values of ses.MNTD ranged from ~-4.5 to −2.0 at elevations 1,707, 1,960, 2,448, and 2,905 m, and then increased to a range of −2.0 to −1. These results suggest that as elevation increased, the soil fungal community became phylogenetically less clustered (Figure 5A). Null model analysis also showed that all βNTI values were <0, indicating significantly lower phylogenetic turnover of soil fungi than expected (Figure 5B). Median βNTI values at 1,707, 1,960, and 2,448 m ranged from −2.5 to −2, suggesting that deterministic processes were driving the soil fungal community at these elevations. At the higher elevations, βNTI values ranged from −2 to −1, indicating that stochastic processes dominated community assembly (Figure 5C). Detailed ecological processes structuring the soil fungal community included homogenous selection at 1,707, 1,960, and 2,448 m, while dispersal limitation was the dominant factor at elevations above 2,746 m. Spearman's correlation analysis showed that Alt, MAP, and MAT had the strongest correlations with ses.MNTD, followed by Wat (Figure 3). For βNTI, TN showed the strongest correlation, followed by Wat and other factors.

## 4 Discussion

### 4.1 Composition and distribution patterns of soil fungal community along the elevational gradients

In this study, the dominant soil fungal phyla on the northern slope of the Central Kunlun Mountains were *Basidiomycota* and *Ascomycota*, which aligns with findings from other mountain ecosystems. However, some subtle differences in the composition of the soil fungal community were observed. Our data revealed that these two phyla, with relative abundances >1.0%, were prominent across the elevational gradients, consistent with previous studies (Tedersoo et al., 2014; Tian et al., 2017; Zhao et al., 2023). However, only these two phyla were observed, which contrasts with findings from other reports (Liu et al., 2018; Ogwu et al., 2019; Ren et al., 2018; Chen et al., 2022). This discrepancy may be attributed to the environmental heterogeneity of the Central Kunlun Mountains, a unique physical geographical subunit of the Qinghai–Tibet Plateau, located at the transition zone between the Tethyan region and the Qinghai–Tibet Plateau (Du et al., 2022). According to Treseder and Lennon (2015), soil fungi can be classified into two functional groups: stress tolerators and decomposers. The dominance of *Basidiomycota* is likely due to their excellent degradation capabilities, supported by a genome rich in genes encoding enzymes for decomposing complex organic matter (Zanne et al., 2019; Baldrian and Valášková, 2008). In contrast, the dominance of *Ascomycota* is likely linked to their tolerance to harsh environmental conditions, with a genome containing a high number of stress tolerance and resource uptake genes (Maestre et al., 2015; Egidi et al., 2019; Opulente et al., 2024). We observed various diversity patterns (unimodal, bimodal, and complex) in the relative abundance of the soil fungal community (Supplementary Figure S3). With increasing elevation, the relative abundance of *Basidiomycota* exhibited a unimodal pattern, which is common in alpine soils (Wang et al., 2015; He et al., 2022). On the other hand, a typical U-shaped pattern was observed for *Ascomycota*, a common trend in microbial elevational distributions (Wang et al., 2022). The contrasting trends in the relative abundances of these two dominant phyla may be linked to their distinct mechanisms of adaptation to harsh environments (Opulente et al., 2024; Treseder and Lennon, 2015).

Our study also found that soil saprotrophs dominated the soil fungal community (Figure 1C), highlighting their crucial role in the ecological processes of the mountain ecosystems. The prevalence of soil saprotrophs followed a clear unimodal pattern, peaking at 2,746 m among the fungal functional guilds. This pattern was similar to that of the class *Agaricomycetes* in the soil fungal community (Figure 1A), which typically functions as saprotrophs, pathogens, and mutualists (Hibbett et al., 2014). According to Yao et al. (2017), most members of Agaricomycetes in soil fungi are oligotrophic and capable of surviving in nutrient-poor environments. Our study revealed that *Agaricomycetes* was one of the most important fungal classes across the entire elevational gradient, consistent with the findings of Eduardo et al. (2018). Many members of this class were categorized as saprotrophs, indicating their significant role in soil carbon cycling on the northern slope of the Central Kunlun Mountains (Ruiz-Dueñas et al., 2020). Ectomycorrhizal fungi, known for their ability to decompose organic matter and compete for nitrogen in the soil, can slow carbon cycling and mediate soil C sequestration in response to climate change (Lindahl and Tunlid, 2015; Averill and Hawkes, 2016). These fungi also have a lower upper growth temperature limit than other fungal guilds (Vetrovsky et al., 2019). In our study, the relative abundance of ectomycorrhizal fungi increased along the elevational gradients (Figure 1C), in line with the findings of Kivlin et al. (2017), suggesting that they form symbiotic relationships with plant roots and exert a stronger influence on ecosystem processes at higher elevations. The contrasting elevational patterns of soil saprotrophs and ectomycorrhizal fungi may reflect their differing responses to changes in climate and soil properties with increasing elevation (Shigyo et al., 2019).

### 4.2 Diversity patterns of soil fungal community along the elevational gradients

Soil fungal elevational diversity patterns vary across multiple factors (McCain and Grytnes, 2010). Environmental heterogeneity, including variations in ecosystems and spatial and temporal scales, can significantly influence the richness and structure of soil fungal communities. Our study revealed that soil fungal α-diversity (Shannon–Weaver index) gradually decreased along the elevational gradients (Figure 2A), suggesting that local climate changes significantly impact soil fungal richness across these gradients. As elevation increases, climate becomes a major limiting factor, restricting the physiological tolerance and resource availability for the species studied. Higher elevations, characterized by harsher climatic conditions, are associated with a reduced species richness of soil fungi on the studied mountain slope. For the two dominant phyla, *Ascomycota* and *Basidiomycota*, their distinct life history strategies, such as varying capabilities to adapt to environmental stresses, likely contribute to their differing patterns along the elevational gradient (Treseder and Lennon, 2015), and distinct preferences for soil nutrients (oligotrophic and copiotrophic, respectively) (Yao et al., 2017). As for the composition of the soil fungal community, an almost increased trend was observed with the increase of elevational gradients (Supplementary Table S2). The trend may be caused by the decline of rare species at higher elevations under harsh environmental stress (Geml et al., 2017). The contrasting trends agreed with the previous reports carried out in almost the same elevational gradients despite the studied mountain ecosystems located in different climate regions (Tian et al., 2017; Shen et al., 2020; Chen et al., 2022). However, the results were not consistent with the studies of Ogwu et al. (2019), Hussain et al. (2021), and Pellissier et al. (2014). The contrasting results suggest that there are no universal elevational diversity patterns for soil fungal communities in that many causes would result in these patterns other than the measured variables (McCain and Grytnes, 2010).

### 4.3 Drivers of the diversity and structure of soil fungal community

Many variables, such as climate, elevation, pH, and nutrients available have been reported to shape the elevational diversity patterns and structure of soil fungal communities (Shen et al., 2014; Liu et al., 2018; Shen et al., 2020). At different scales, the effects of environmental variables on the abundance and richness of soil fungi often vary on the niche conditions in mountain systems. For example, Talley et al. (2002) found that the abundance and richness of fungi in a habitat were limited by the duration of unfavorable weather conditions. Tedersoo et al. (2014) found that climate factors (MAP and MAT) at a global scale strongly affected the fungal richness and functional groups. What's more, changes in substrate quality and availability also may result in shifts in fungal abundance (Allison et al., 2007; Bossuyt et al., 2001; Frey et al., 2004). A rise of altitude is often related to an increase in environmental harshness (Körner, 2007), which greatly affects soil fungal richness and abundances along mountain elevational gradients (Margesin et al., 2009; McCain and Grytnes, 2010). In this study, MAP significantly increased with the increase in elevational gradients, whereas MAT decreased. Spearman's correlation analysis revealed that altitude, MAP, and MAT were the strongest predictors of soil fungal α-diversity (Figure 3). The causes may be related to the change of the altitude, resulting in the fluctuations of other gradients (abiotic and biotic factors), which would greatly impact species richness and diversity distribution patterns (McCain and Grytnes, 2010). Among these gradients, climate often provides physiological limits of the minimum and maximum niche values to species, which greatly impact on species richness (McCain and Grytnes, 2010). The results were consistent with previous reports (Tedersoo et al., 2014; Vetrovsky et al., 2019; Shen et al., 2020; Tian et al., 2017).

Among the environmental factors, soil water has a critical role in governing soil biodiversity and function (Zhang et al., 2023; Schimel, 2018), especially in arid ecosystems (Maestre et al., 2015; Berdugo et al., 2020). In the mountain ecosystems, precipitation is mainly generated from the changes of the elevational gradients, especially those located in drylands (Körner, 2007; Viviroli and Weingartner, 2004). Although Spearman's analysis showed that many variables significantly correlated with soil fungal α-diversity (Figure 3), PLSPMs indicated that Wat was the strongest environmental factor (Figure 4C). Interestingly, the mode of Wat on the species richness in soil fungal community varied on the elevational gradients (Figure 4, Supplementary Table S5). At the lower elevational gradients (≤2,448 m), Wat influenced soil pH directly, which then impacted the species diversity of soil fungi (Figure 4A). Moreover, at the higher elevational gradients (≥2,746 m), Wat reached a certain threshold and then affected the species diversity of soil fungal abundance directly (Figure 4B). Soil pH has been confirmed as one of the main factors shaping soil fungal diversity (Liu et al., 2018; Wang et al., 2015). A study by Slessarev et al. (2016) showed that small changes in soil water balance would cause a steep transition from alkaline to acid soils across natural climate gradients. The changes in soil pH would impact the availability of soil nutrients and soil properties (Barrow and Hartemink, 2023). We inferred that there may be a water content threshold, which would regulate soil pH and later influence soil fungal α-diversity. Despite soil water as the only factor significantly correlated with the fungal community composition across the whole transect (Figures 2B, 3, Supplementary Tables S3, S4), PLSPMs indicated Veg was the most important variable in structuring soil fungal community (Figure 4). In fact, Veg was significantly impacted by Wat, meaning that the effects of Wat on soil fungal structure were mainly indirect. Despite the non-significance of Wat in driving soil fungal community composition in PLSPM analysis, dbRDA and MRM analysis revealed that Wat was one of the main factors affecting the composition of the fungal community (Supplementary Tables S3, S4). The results support our first hypothesis. Our data did not fully align with the findings of Jiao et al. (2021), which may be attributed to the distinct, multifaceted effects of elevation-related gradients along the Central Kunlun Mountains, as well as differences in the regional scales considered in both studies. Although soil nutrients, such as nitrogen and carbon, are known to significantly influence the composition of fungal communities (Yang et al., 2022; Lauber et al., 2008), our study revealed that Veg was the strongest factor in structuring the fungal community (Figure 4, Supplementary Table S5). This finding contrasts with the results for soil fungal α-diversity. The difference may be due to the fact that the initial composition of the soil fungal community is largely driven by nutrients from the plant community, including plant litter and metabolites entering the soil environment. In contrast, the species richness of the soil fungal community is more strongly influenced by environmental and climatic conditions that support growth. For soil biota, plant litter serves as a primary source of organic substrates entering the soil (Waldrop et al., 2006). However, differences in litter composition, such as cellulose and lignin contents, would result in changes in the community composition of decomposers (Sterkenburg et al., 2015) because some main fungal taxa (such as *Basidiomycota* and *Ascomycota*) have different patterns for lignin degradation and oxidative enzyme secretion (Liers et al., 2011). What's more, roots can secret many metabolites to the rhizosphere (Vives-Peris et al., 2020), which would greatly influence the fungal community. Prober et al. (2014) reported that plant β-diversity was significantly correlated with the β-diversity of fungal communities in temperate-zone grasslands. A similar study by Chen et al. (2017) found that fungal community composition was primarily shaped by plant community composition in arid and semi-arid temperate grasslands. Based on these findings, it is not surprising that the β-diversity of the soil fungal community increased with higher plant diversity on the northern slope of the Central Kunlun Mountains. This suggests that plant diversity plays a key role in influencing the structure and variability of soil fungal communities, particularly in regions with varying climatic and ecological conditions (Gui et al., 2010). Our results agreed with the studies of Prober et al. (2014) and Chen et al. (2017).

Interestingly, the modes in the effects of Veg on the composition of soil fungi community changed along with the increase in elevational gradients. At the lower transect (≤2,448 m), above-ground vegetation adjusted their rhizosphere environments (nutrients and physical–chemical properties; Figure 4D), which shaped the composition of soil fungi. In addition, there was a possibility that soil water content impacted the soil fungal community via pH regulation (Slessarev et al., 2016). At higher elevation sites, above-ground vegetation had a direct influence on soil fungal community composition (Figure 4E). Across the entire transect, vegetation (Veg) impacted soil fungal community composition through two pathways: directly and indirectly, through its effects on soil pH (Figure 4F). This can be attributed to nutrients, such as litter and root exudates, provided by plants, as well as their ability to regulate soil environments, including pH and physical factors (Waldrop et al., 2006; Vives-Peris et al., 2020). The strong correlation between vegetation coverage and soil fungal community composition supports our second hypothesis, highlighting the vital role of vegetation in mountain ecosystems. Other factors, such as soil pH and salinity (Sal), may also contribute to the composition of soil fungal communities (Figures 2B–4).

### 4.4 Ecological processes shaping soil fungal community construction

Phylogenetic diversity measure is of great importance to predict ecosystem functions and promote ecosystem stability (Cadotte et al., 2012; Srivastava et al., 2012). With the increase in elevational gradients, soil fungal communities are often phylogenetically clustered (Mikryukov et al., 2023), which is also observed in soil fungal guilds (Egan et al., 2017). Our study found that the soil fungal community exhibited a less clustered phylogenetic trend with the increase in the elevational gradients (Figure 5A). According to the views of Mikryukov et al. (2023), the observed trend may be linked to the impact of rapid speciation in specific fungal taxa in colder climates, which influences the phylogenetic structure of soil fungal communities. Environmental filters, such as climate gradients, soil nutrient availability, and soil water content, are the primary processes shaping soil fungal communities. For instance, arbuscular mycorrhizal fungi are often found to be clustered in natural ecosystems, reflecting their adaptation to specific environmental conditions. These filters drive the distribution and diversity of fungal species, particularly in response to temperature and moisture variations (Chen et al., 2017). In this study, altitude and MAP showed the highest positive correlation values with ses.MNTD, whereas MAT had the lowest correlated value with ses.MNTD (Figure 3). The drastic changes in climate and other climate-related gradients (Supplementary Figure S2) within the region studied may result in rapid speciation and phylogenetic clustering along the increase in elevational gradients (McCain and Grytnes, 2010; Mikryukov et al., 2023).

It is very important to know how ecological processes influence the assembly of the soil fungi community, which would help us learn the underlying mechanisms and their responses to global environmental change (Peay et al., 2016; Stegen et al., 2012). Our data showed that the phylogenetic turnover of the soil fungal community was less than the expected phylogenetic turnover with the increase in the elevational gradients (Figure 5B). At the sites lower than 2,448 m, βNTI <-2 indicated that the phylogenetic turnover of soil fungal community was governed by homogeneous selection (Stegen et al., 2013). According to Stegen et al. (2015), homogeneous selection can dominate if communities occur in the same selective environment, which is relatively strong. While at the sites higher than 2,746 m, stochastic processes dominated the assemblage of soil fungal community (Figure 5B) (Stegen et al., 2013). Dispersal limitation was primarily responsible for the phylogenetic turnover of the soil fungal communities at the sites higher than 2,746 m (Figure 5C). The causes may be related to no direct dispersal occurring between the soil fungal communities at the sites higher than 2,746 m (Stegen et al., 2015). The results do not support our third hypothesis. The reasons may be related to environmental filtering and spatial heterogeneity of the region studied. A study by Kivlin et al. (2014) showed that soil fungal communities were mostly assembled by environmental filtering. Our data revealed soil TN exhibited the highest correlated value with βNTI (Figure 3). Although TN increased with the increase in elevational gradients, the available nitrogen may be lower at higher elevational gradients due to the decreases in microbial N metabolite rates in colder ecosystem (LeBauer and Treseder, 2008; Pajares and Bohannan, 2016), and the stronger competition for nitrogen between soil fungi and plant with higher coverage and diversity (Supplementary Figure S2) (Kuzyakov and Xu, 2013; Gui et al., 2010).

For soil fungal communities, the strongest factors affecting their survival and growth may vary with the increase of elevational gradients (Supplementary Tables S3–S5, Figure 4). This research confirmed that the influence of stochastic assembly processes increases with the decrease in fungal richness (Stegen et al., 2012; Peay et al., 2016; Jiao et al., 2021). However, our result was inconsistent with the study of Hussain et al. (2021), who postulated that stochastic processes dominate in the community assemblage of soil fungi along the elevational gradients (889–3,837 m). The reasons may be attributable to the environmental heterogeneity of the region focused (Du et al., 2022). Another study indicated that dispersal limitation was the primary ecological process in structuring soil fungal communities in alpine grasslands along elevational gradients (2,813–5,228 m) (Li et al., 2023), which aligns with our findings that dispersal limitation dominated the soil fungal community at altitudes higher than 2,746 m (Figure 5C). However, our data did not fully support our third hypothesis. The contrasting results suggest that the influence of deterministic and stochastic processes on soil fungal community assembly is elevationally dependent. Additionally, other unmeasured abiotic and biotic factors specific to the studied regions likely contribute to the composition of the soil fungal community (Stegen et al., 2015). The variations in ecological processes may be related to the changes in the main environmental factors, which affect soil fungal communities in elevation-dependent manners.

## 5 Conclusion

We characterized the elevational diversity patterns of the soil fungal community on the northern slope of the Central Kunlun Mountain ecosystems across elevational gradients (1,707–3,548 m) and identified the related ecological mechanisms. Diverse elevational patterns were observed in the soil fungal community, fungal guilds, and taxa. Along the elevational gradient, soil fungal α-diversity exhibited a decreasing trend, whereas β-diversity showed an increasing trend. Spearman's correlation analysis revealed that altitude and climate (MAP and MAT) were the strongest predictors for soil fungal α-diversity. PLSPM analysis indicated that soil water was the most important variable driving soil fungal α-diversity, while vegetation coverage and soil pH were the main factors significantly correlated with the composition of the soil fungal community. Across the elevational gradient, soil water influenced fungal α-diversity in both an indirect and direct manner, responding to the transition from lower elevations (≤2,448 m) to higher elevations (≥2,746 m). Vegetation coverage, significantly influenced by soil water, was the primary factor affecting the composition of the soil fungal community. Null model analysis revealed that homogeneous selection and dispersal limitation dominated soil fungal community assembly at elevations below 2,448 m and above 2,746 m, respectively. These results contribute to advancing our understanding of soil fungal diversity patterns in mountain ecosystems, particularly in arid and ecologically fragile regions, and how these systems respond to climate change.

## Data Availability

The datasets presented in this study can be found in online repositories. The names of the repository/repositories and accession number(s) can be found in the article/Supplementary material.
