# Supplementary figures and images for: Effects of soil water on fungal community composition along elevational gradients on the northern slope of the Central Kunlun Mountains
Source: Front Microbiol. 2025 Jan 8;15:1494070. doi: 10.3389/fmicb.2024.1494070 (PMC11753354; doi:10.3389/fmicb.2024.1494070)

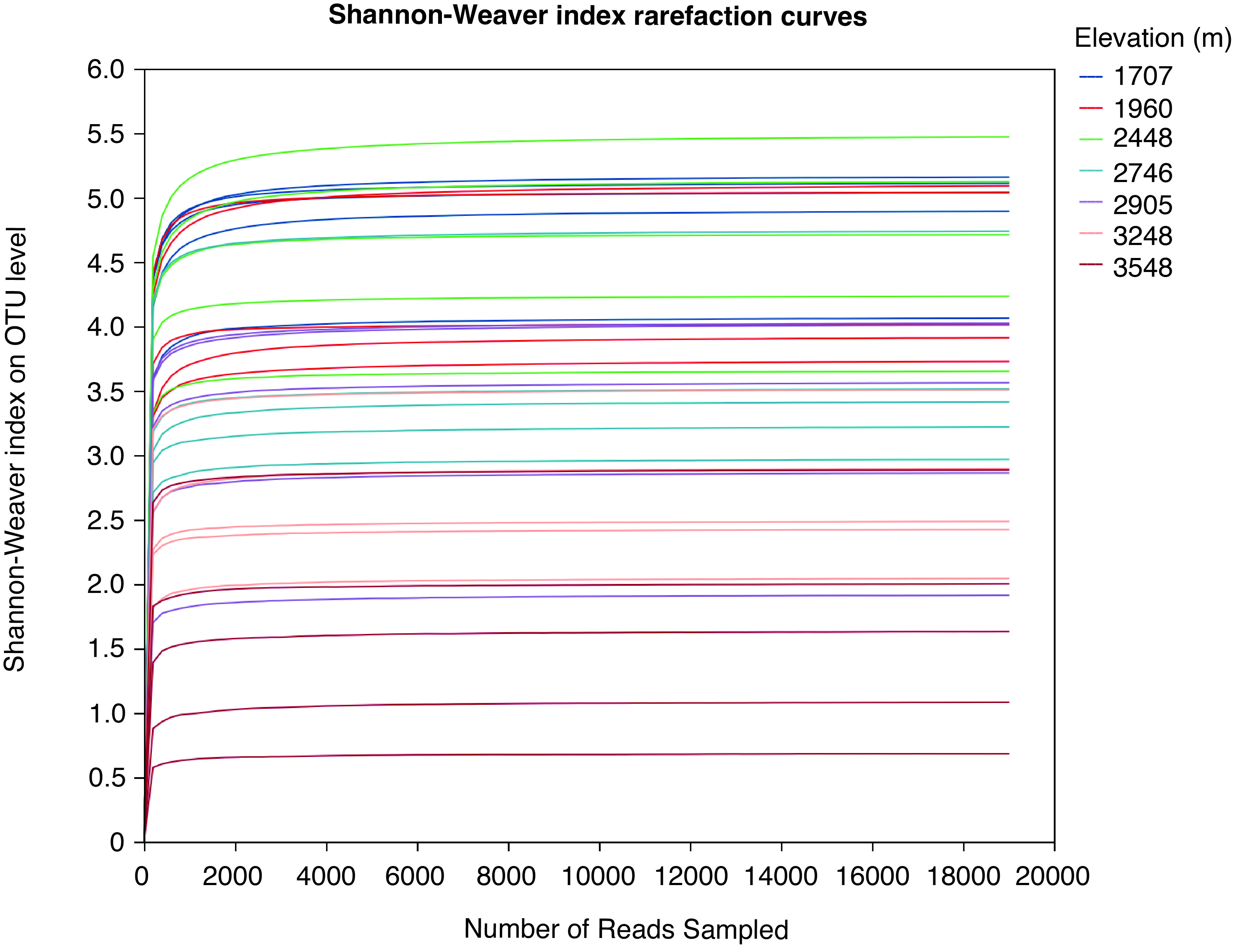

Supplement: Supplementary file 2 [file Image_1.jpeg]

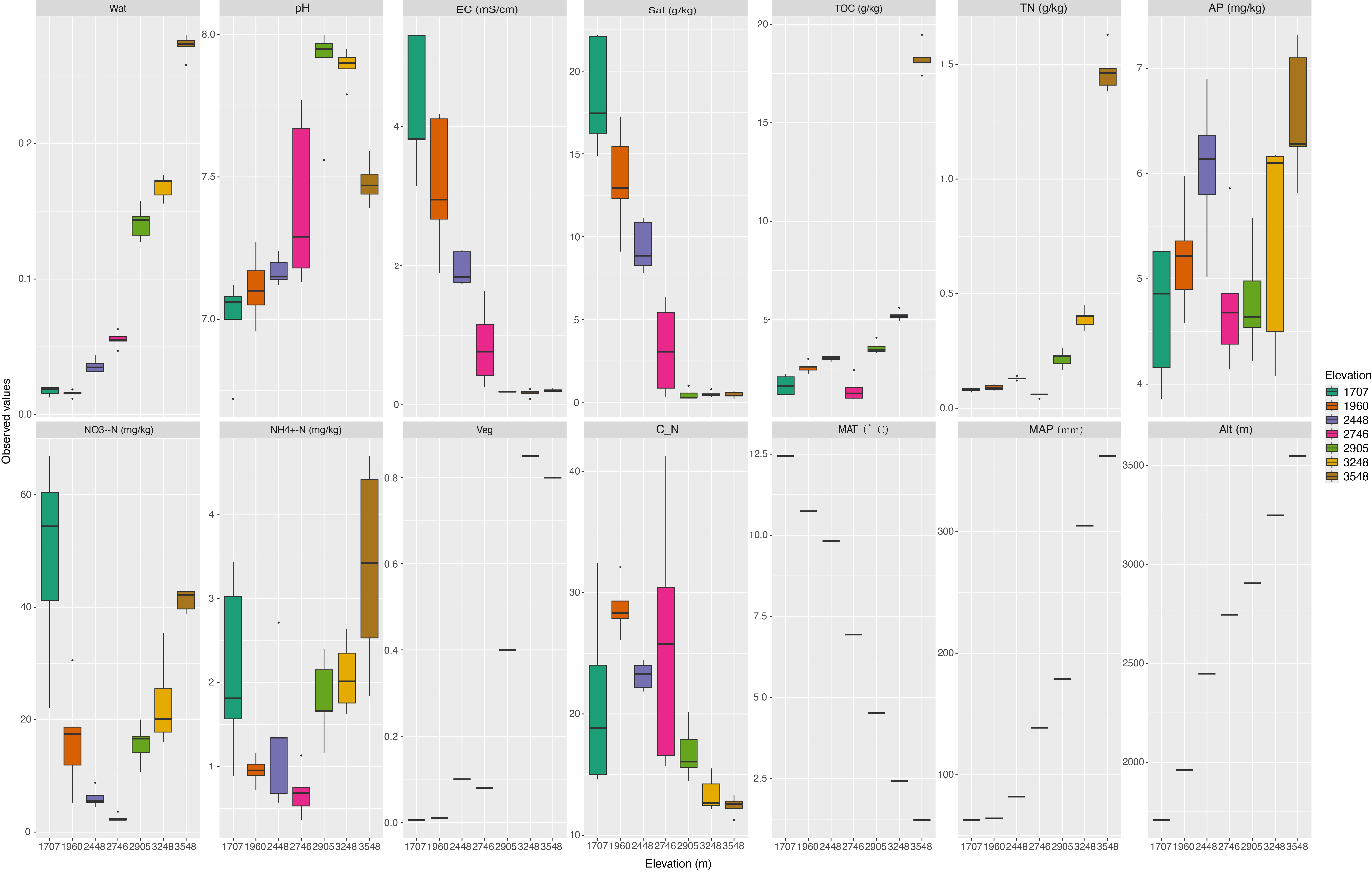

Supplement: Supplementary file 3 [file Image_2.jpeg]

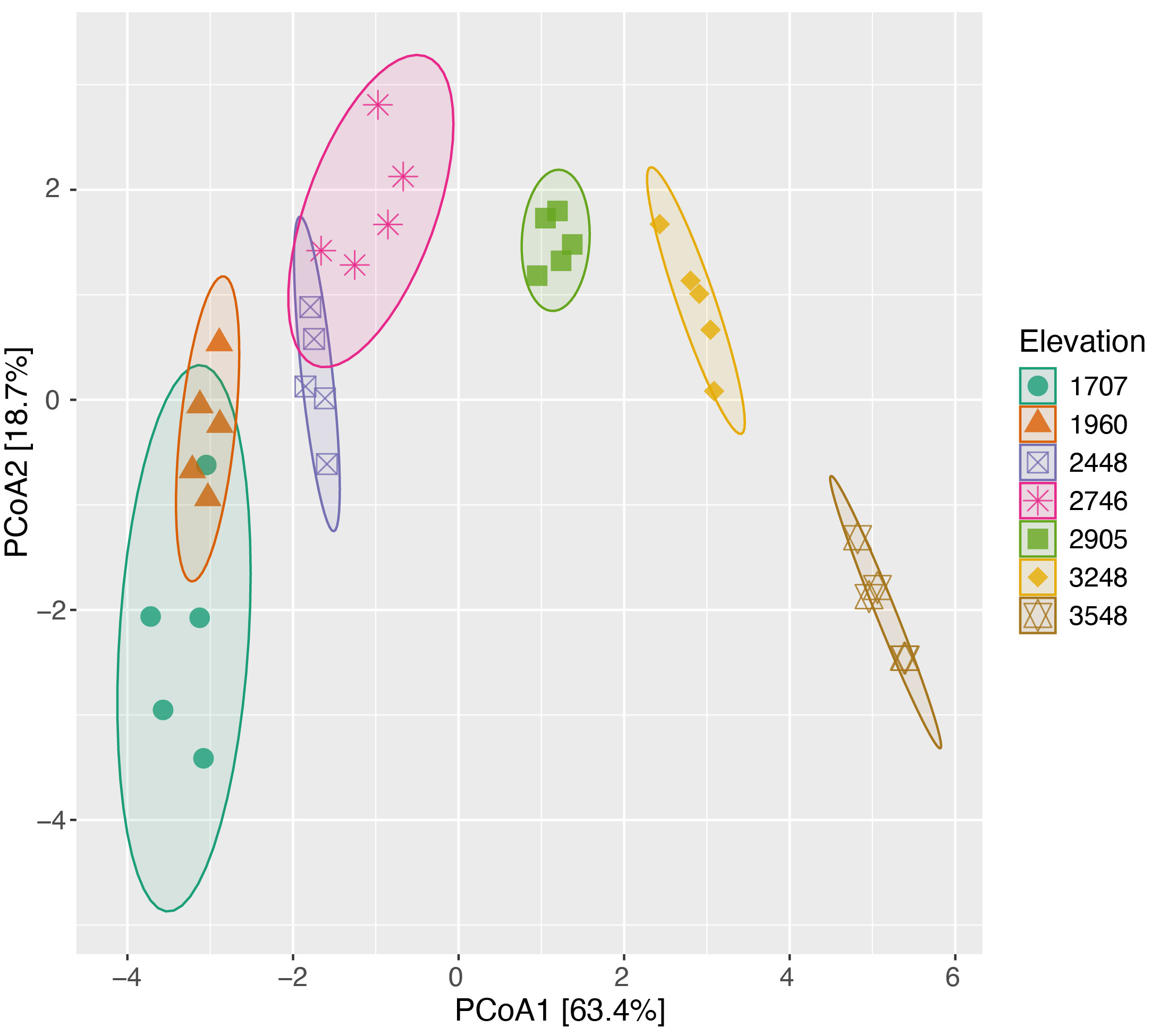

Supplement: Supplementary file 4 [file Image_3.jpeg]

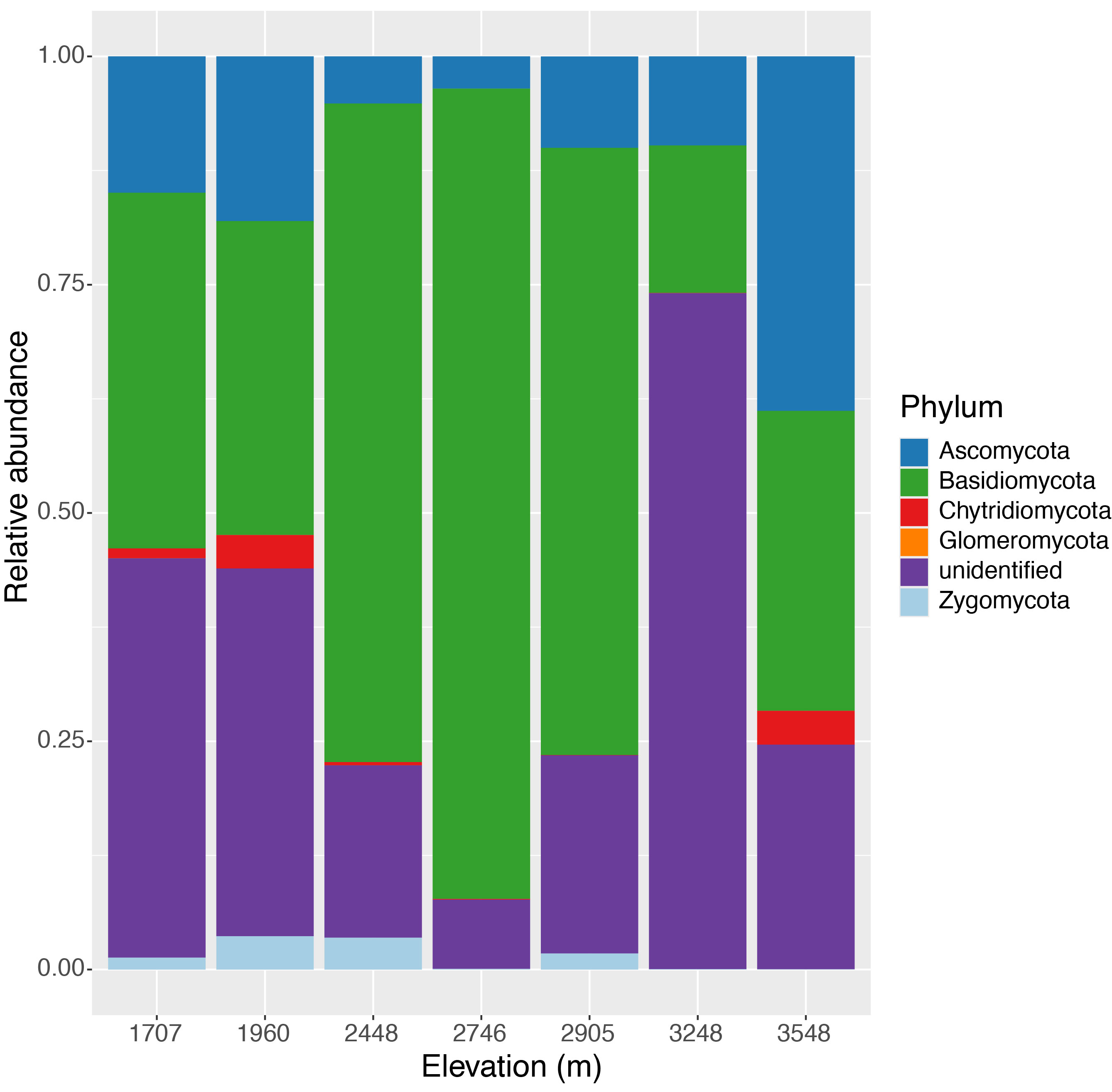

Supplement: Supplementary file 5 [file Image_4.jpeg]
